# Flourishing levels among health and non-health profession students in Saudi Arabian colleges

**DOI:** 10.3389/fpsyt.2024.1469845

**Published:** 2024-10-25

**Authors:** Emad Shdaifat, Amira Alshowkan, Friyal Alqahtani, Hoda Alebiary, Mona AL-Qahtani, Nagla Alsaleh, Neama Kamel

**Affiliations:** Community Health Nursing, College of Nursing, Imam Abdulrahman Bin Faisal University, Dammam, Saudi Arabia

**Keywords:** flourishing, public health, religion, Saudi Arabia, students, universities, well-being

## Abstract

**Objectives:**

This study aimed to evaluate the levels of flourishing among university students, compare these levels between students in health-related and non-health-related colleges, and identify factors that predict flourishing.

**Methods:**

A cross-sectional study was conducted at Imam Abdulrahman Bin Faisal University, Saudi Arabia, involving 1,148 students from the first to fourth year across both academic sectors. Data were collected utilizing the self-reported Global Flourishing Study Questionnaire (GFS) during the period from September 2023 to June 2024. Multistage sampling techniques were employed to ensure a representative sample, with data collection facilitated through a self-administered electronic link on QuestionPro resulting in a response rate of 51.7%. Data analysis was performed using SPSS version 22, incorporating descriptive statistics, t-tests, ANOVA, and regression analysis to identify predictors of flourishing. The reliability and validity of the questionnaire were assessed using Cronbach’s alpha and Pearson’s correlation analysis.

**Results:**

The study included nearly equal proportions of students from health (51.0%) and nonhealth (49.0%) colleges. The average flourishing score was 85.6 (SD=18.6), with a slight increase in health colleges (85.7) compared with non-health colleges (85.4). Factors that significantly affected flourishing included gender, employment status, exercise frequency, experiences of abuse, and income. In the multiple regression analysis, well-being emerged as the strongest predictor, followed by external factors, disposition, and behavior. Gender exhibited a positive association with flourishing, whereas religion had a negative influence. Furthermore, employment and higher income levels were found to positively contribute to flourishing.

**Conclusion:**

This study revealed elevated levels of flourishing among university students in Saudi Arabia, with a notable average score of 85.6. Although the differences between students enrolled in health-related and non-health-related colleges were minimal, significant predictors of flourishing were identified, including well-being, external factors, disposition and behavior, gender, religious affiliation, employment status, income, frequency of exercise, and experience of abuse. These findings underscore the complexity of flourishing and highlight the necessity of considering a range of sociodemographic and lifestyle factors to promote student well-being.

## Introduction

Well-being includes multiple personal state dimensions, including physical, emotional, and social. College students have been found to have increased levels of psychological and academic distress, which commonly alters their mental well-being (1, 2). Previous studies comparing the mental health of college students in health and non-health colleges reported inconsistent results. For instance, a study comparing the prevalence of depressive symptoms among students from medical, dental, and engineering colleges found that depressive symptoms were present in 40.3%, 38.5%, and 34.7% of engineering, dental, and medical students, respectively (3). Another study found that the prevalence of depression among medical and engineering students is 20.6% and 15.3%, respectively. Additionally, female students in non-health colleges reported more stress than did those in health colleges (4, 5). Thus, studying the influences that impact flourishing levels among students enrolled in health and non-health colleges, and the related factors in Saudi Arabia, is valuable.

Flourishing and well-being have been used in the field of positive psychology. While well-being is composed of hedonic concepts (happiness and life satisfaction) and eudaimonic concepts (e.g., having meaning in life and positive relations), flourishing refers to the ideal state of functioning in all aspects of life (6, 7). The flourishing concept is based on the theory of PERMA’s well-being, which focuses on five fundamental elements: positive emotion, engagement, relationship, meaning, and accomplishment (8). The combination of these five elements results in personal flourishing. The study of the PERMA model among undergraduate students is vital because they experience unstable mental health during their life transition, which may affect their achievement of optimum well-being (9).

Flourishing is an expression of emotional state, psychosocial operation, and social well-being and is viewed as a continuum of mental health concepts (10). A positive relationship was observed between psychological health and physical health. Psychological well-being alone does not achieve the comprehensive meaning of the flourishing concept. Thus, flourishing is attained as a result of individual reports that all life aspects are good (11). As flourishing incorporates positive well-being and mental health, quality of life is a concept of predominantly feeling positive well-being (12). Hence, quality of life can be considered as the cornerstone of individual flourishing. Positive mental health among college students is associated with academic achievement, optimal health, and positive social parameters (13). Nonetheless, there are few studies on the levels of flourishing and its predictors among university students, specifically those in health and non-health fields.

Studies among university students have claimed that flourishing enhances students’ lives with and without disease symptoms. For students with symptoms, work flourishes to avoid mental illness (14). In addition, it promotes positive consequences concerning academic affirmation and community engagement (13). Easing flourishing among students helps them achieve their academic and career goals without interruption (15).

Similarly, positive psychological interventions are valuable and help retain students (16). According to Ahlstedt et al. (42) employees who flourish in their jobs are the intended outcome. Additionally, they discovered that motivating nurses through daily communication positively affects their flourishing. A study conducted in Cyprus by Sürücü et al. (17) emphasized that fear may negatively impact flourishing. This is evident during the COVID-19 pandemic as people are segregated as fearful and anxious, resulting in negative mental health consequences. Social connections are an influential domain in individual flourishing, and have been restricted by pandemics. In addition, financial uncertainty is negatively influenced.

**Figure 1:**
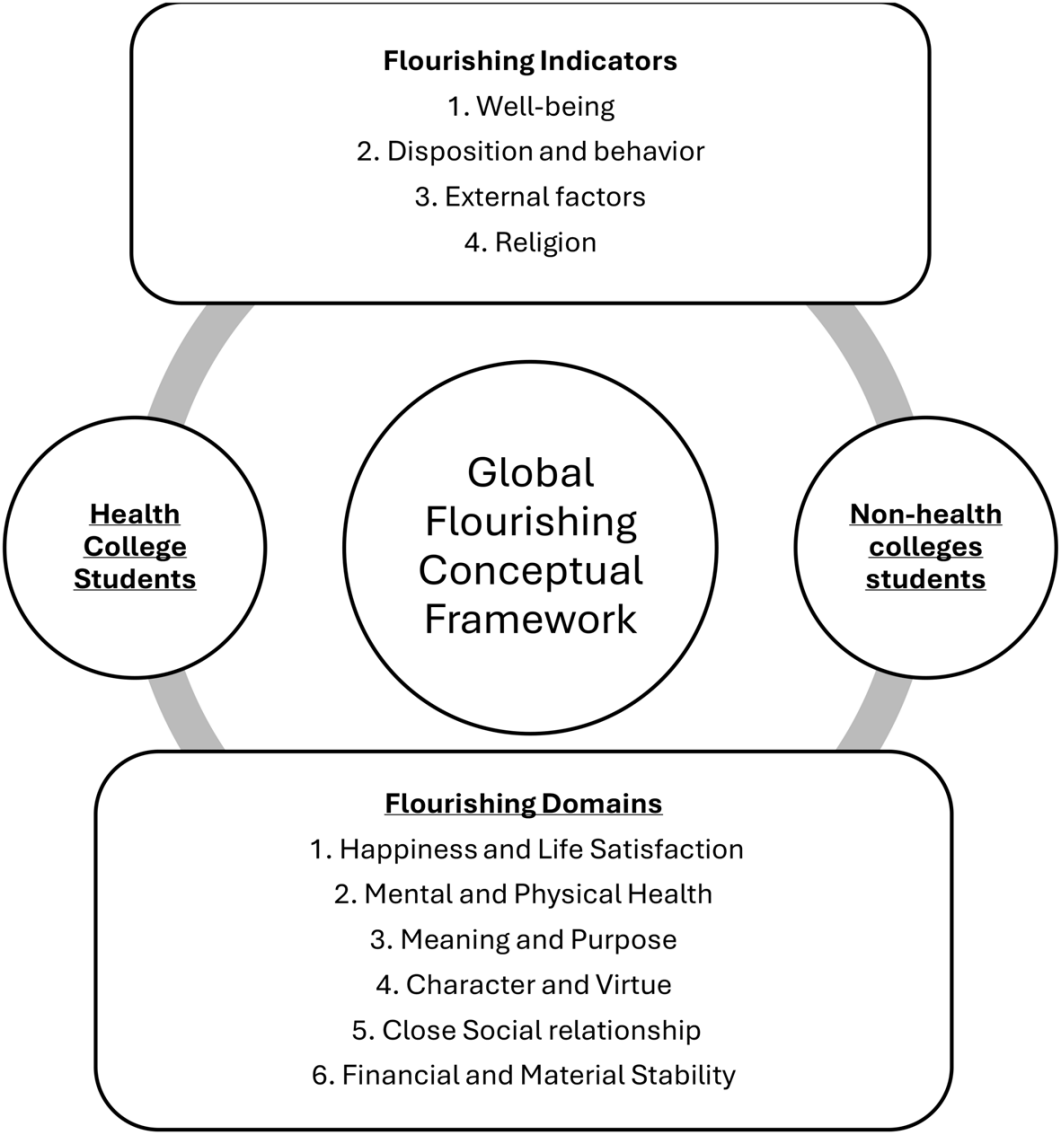
Global flourishing conceptual framework based on Crabtree (18).

In an extensive review of the literature on flourishing, we found insufficient studies specifically on undergraduate students enrolled in health and nonhealth colleges. Therefore, this study aims to determine the level of flourishing among university students. In addition, it compares flourishing levels between college students enrolled in health and non-health colleges. Furthermore, this study aimed to determine predictors of flourishing among university students. Thus, strategies for enhancing mental health are suggested to effectively facilitate student flourishing. Studying the dynamics between the six flourishing domains simultaneously within a comprehensive framework of joint flourishing concepts can offer a vital understanding of the effect of each domain in prompting an inclusive flourishing conceptual framework, as shown in **Figure 1** (18). These findings can serve as indicators of public health and promote well-being.

## Methods

### Site, setting, and design

This cross-sectional study was conducted at Imam Abdulrahman Bin Faisal University, Saudi Arabia. The study included students from both health- and non-health-related professional programs. Multistage sampling techniques were used to ensure representativeness of the sample.

### Sampling and sample size

Students in their first to fourth years of study enrolled in both health and non-health professions were included in the study. Data were collected between September 2023 and March 2024 using a self-report questionnaire. In order to be included, students needed to express their willingness to participate, while those on academic probation, on leave, or unwilling to participate were excluded from the study. To establish the sample size for comparing the means of two independent groups, namely health and non-health professional students, we conducted an *a priori* power analysis using t-tests to examine the differences between the two means. The objective of this analysis was to detect a small effect size (d = 0.2) with a two-tailed test, alpha error probability (α) of 0.05, and desired power (1-β) of 0.80. The calculations yielded a non-centrality parameter (δ) of 2.8071, critical t-value of 1.9630, and 786 degrees of freedom. Consequently, the determined sample size for each group was 394, resulting in a total sample size of 788 students and achieving an actual power of 0.80. Ultimately, the study included 1,148 students to ensure robust and reliable outcomes, thus surpassing the required sample size.

A multistage sampling technique was used to improve the validity of the study findings and to achieve a higher level of sample representativeness. The population was initially stratified according to profession type, distinguishing between health and non-health professions. Within each stratum, participants were selected based on their educational level, employing a systematic approach aimed at achieving balanced representation across all levels.

### Ethical considerations

The study received ethical approval (IRB-2022-04-531) from the Institutional Review Board at Imam Abdulrahman Bin Faisal University, Saudi Arabia. Detailed information sheets were provided to the participants to outline the objectives, significance, and potential benefits of the study. Implicit consent was obtained from all participants, guaranteeing that their participation was voluntary, and that their responses remained anonymous. Measures have been implemented to safeguard data confidentiality and security. Participants were informed of the potential risks and precautions taken to minimize them. Contact information was provided to address any inquiries or concerns related to this study.

### Description of measurement tool

Data were obtained using the Global Flourishing Study Questionnaire (GFS) to evaluate the level of flourishing and its determinants. Flourishing, measured using the Secure Flourish Index (SFI), includes six domains: happiness and life satisfaction, mental and physical health, meaning and purpose, character and virtue, close social relationships, and financial and material stability. Participants were required to rate each item on a 10-point Likert scale, ranging from 0 to 10. Each domain accounted for a maximum of 20 points, resulting in a total score of 120. Higher scores indicate a greater level of flourishing. Each domain was examined using two representative items: overall life satisfaction for the happiness domain, and a rating of physical health for the mental and physical health domains. This structured methodology facilitated a comprehensive assessment of participants’ well-being (11, 19).

The questionnaire also included inquiries regarding numerous determinants of flourishing, including self-reported well-being, disposition and behavior, external factors, religion/spirituality, and demographic variables. The self-reported well-being component encompasses dimensions such as life satisfaction, contentment, and a sense of equilibrium or concord, thereby reflecting overall happiness and self-assessment of mental and physical health. The scores for each determinant were derived by aggregating responses within each category.

Determinants pertaining to disposition and behavior encompass personal traits, forgiveness, self-assurance, optimism, lifestyle choices (such as alcohol and tobacco consumption), and physical activity. External factors include satisfaction with friendships and relationships, societal trust, availability of support networks, a sense of community belonging, confidence in the government, and experiences of discrimination. These factors shed light on individuals’ social interactions, trust levels, support systems, sense of belonging, perceptions of government entities, and discrimination. Religion and spirituality involve connections to higher powers, spiritual beliefs, and participation in communal practices, which provide individuals with meaning, guidance, and a sense of belonging (11). Sociodemographic and lifestyle factors such as gender, age, marital status, working status, income, smoking, exercise, experiences of abuse, and college affiliation were also considered. The questionnaire employed various formats, including 1-to-10 scales, Likert scales (ranging from “always” to “never”), yes/no queries, and scales ranging from “many times” to “never” and “a lot” to “never.” Examples of the questions included “freedom in life,” “remaining hopeful about the future,” and “connection to religion.” The determinants were assessed as follows: well-being (determinant 1) encompassed 14 questions related to life satisfaction, content, and mental and physical health. Disposition and behavior (Determinant 2) were evaluated using six questions that focused on hope and gratitude. External factors (Determinant 3) were gauged using nine questions on loneliness and participation in group activities. Religion (determinant 4) was examined using six questions pertaining to religious or spiritual connections. These questions were based on the GFS link (18).

### Validity and reliability

The SFI, as indicated by Weziak-Bialowolska et al. (19), has been found to have strong psychometric properties. These properties include indices such as CFI = 0.978, TLI = 0.971, RMSEA = 0.041, SRMR = 0.026, and an internal consistency of 0.86. Validation of the GFS was further supported by (20), who confirmed the scale’s precision and reliability through content evaluation. The evaluation was conducted by a panel of four doctoral nurses and two psychologists. The Arabic version of the GFS was used to assess flourishing levels among the university students. Confirmatory Factor Analysis (CFA) was conducted in Arabic using AMOS, and the results showed a satisfactory model fit. The measures were CMIN/df = 2.138, GFI = 0.960, CFI = 0.962, SRMR = 0.040, and RMSEA = 0.059. These results are consistent with established standards and confirm the scale’s robust psychometric properties (21–24). Therefore, this scale can be considered valid for measuring the flourishing levels.

**Table 1** presents the results of Pearson’s correlation analysis for the total scores of each determinant and their respective items, including the flourishing scale. The correlation coefficients for the flourishing scale ranged from 0.458 to 0.734, indicating moderate-to-strong correlations. Cronbach’s alpha for the flourishing scale was 0.838. The well-being determinant exhibited coefficients ranging from 0.377 to 0.720, with Cronbach’s alpha of 0.824. For disposition and behavior, the coefficients ranged from 0.849 to 0.861, with Cronbach’s alpha of 0.631. The external factor determinant showed coefficients ranging from 0.219 to 0.716, with a Cronbach’s alpha of 0.546. Lastly, the religion determinant has coefficients ranging from 0.546 to 0.725, with a Cronbach’s alpha of 0.692 (25).

**Table 1:** Person correlation analysis results between flourishing scale and the determinants.

Scale & Determinants	r Value	Cronbach’s Alpha	Critical value
Flourishing scale	0.458 to 0.734	0.838	0.0579
Well-being	0.377 to 0.720	0.824	0. 0579
Disposition and behavior	0.849 to 0.861	0.631	0. 0579
External factors	0.219 to 0.716	0.546	0. 0579
Religion	0.546 to 0.725	0.692	0. 0579

### Data collection procedure

After receiving ethical approval, the primary investigator collaborated with course coordinators to gather data. The students were provided with comprehensive information regarding the aims, methodologies, and protocols of the study with emphasis placed on voluntary involvement and confidentiality. Invitations comprising a survey link and barcode were disseminated through WhatsApp to minimize face-to-face interactions and mitigate the potential influence of coordinators. This strategy, administered by team leaders, ensured transparency concerning participation and safeguarded the ethical principles. Anonymity was preserved by omitting the identifiable information. The survey, which required approximately 15-20 minutes to complete, yielded a response rate of 51.7%. Surveys were distributed using the online platform QuestionPro (www.questionpro.com), and authorization to use the questionnaire was obtained from the original authors.

### Data analysis

Data were stored and analyzed using the Statistical Package for Social Sciences (version 22). Categorical data were presented as frequencies and percentages, whereas continuous data were reported as means, standard deviations, and ranges. To compare students’ scores across the six domains of flourishing, t-tests and one-way ANOVA were conducted, considering demographic variables and other related data. Cronbach’s alpha was used to assess internal consistency of the items. Correlations between study variables were evaluated using correlation coefficients, and regression analysis was performed to identify predictors of flourishing. Statistical significance was set at P < 0.05.

Several measures were implemented to enhance the reliability and validity of the findings. No data were missing. The response rate was deemed satisfactory at 51.7%, indicating a high level of participation. Outliers were identified and subsequently removed by applying a Mahalanobis distance threshold of 25.260 (α = 0.05, n = 1150), resulting in the removal of two participants. This threshold, derived from the chi-square distribution, adheres to the established statistical significance criteria and ensures accurate detection of outliers. Stepwise regression analysis was performed to identify the predictors of student flourishing, considering demographic variables (gender, age, marital status, employment status, income, and education) and determinants (well-being, external factors, religion, disposition, and behavior). Flourishing levels were categorized into three groups: low (≤40), medium (41–80), and high (>80).

## Results

**Table 2** highlights the key demographic characteristics of the 1,148 participants. Females comprised the majority of the study population (72.5%). A significant proportion (56.4%) of the participants were aged 20 years or younger. Regarding university affiliation, almost equal distributions of 51.0% and 49.0% were observed between health and non-health universities. In addition, a clear majority (91.8%) of participants were unemployed. Regarding income, 45.6% of the participants earned more than 12,000 SAR and 95.1% were non-smokers.

**Table 2:** Demographic and lifestyle characteristics of study participants (n=1148).

Variable	Categories	Frequency	Percent
Gender	Male	316	27.5
	Female	832	72.5
Age	≤ 20	648	56.4
	> 20	500	43.6
Marital Status	Single	1048	91.3
	Married	100	8.7
College	Health	586	51.0
	Non-health	562	49.0
Working Status	Working	94	8.2
	Not Working	1054	91.8
Income (SAR)	< 3000	183	15.9
	3000-12000	442	38.5
	> 12000	523	45.6
Smoking	Nonsmoker	1092	95.1
	Smoker	56	4.9
Exercise	No Exercise	472	41.1
	≤ 3 times/week	437	38.1
	> 3 times/week	239	20.8
Abused	Yes	267	23.3
	No	779	67.9
	Not Sure	102	8.9

**Table 3** shows the flourishing scores among participants categorized by health and non-health colleges as well as an overall student. The overall average flourishing score was 85.6 (SD=18.6) for health colleges was 85.7, and 85.4% for nonhealth colleges. High flourishing was prevalent among all students (63.3% overall, 65.9% in health colleges, and 60.7% in non-health colleges).

**Table 3:** Flourishing levels among all students and by college type.

Descriptive	All	Health	Non-Health
Mean (SD)	85.6 (18.6)	85.7 (18.5)	85.4 (18.6)
Range	96	95	96
Median (IQR)	87.0 (25.0)	88.0 (25.0)	86.0 (26.0)
Levels	Frequency (Percent)	Frequency (Percent)	Frequency (Percent)
Low (≤ 40)	17 (1.5)	10 (1.7)	7 (1.2)
Medium (41-80)	404 (35.2)	190 (32.4)	214 (38.1)
High (>80)	727 (63.3)	386 (65.9)	341 (60.7)

SD, Standard Deviation, IQR, Interquartile Range.

**Table 4** illustrates the significant differences in flourishing levels based on gender, employment status, exercise, abuse experience, and income. Males showed higher flourishing than females in all groups (P < 0.001). Working participants recorded higher well-being than non-working individuals, with significance in both the overall and non-health categories (P = 0.005). Those who exercised more than three times per week demonstrated higher flourishing (P < 0.001). Participants who reported abuse had lower flourishing scores (P < 0.001). Furthermore, higher income (>12,000 SAR) was associated with increased flourishing, particularly in health colleges (P < 0.001). **Table 5**

**Table 4:** Comparative analysis of flourishing determinants across health and non-health colleges (N=1148).

Variables		ALL			Health			Non-Health		
Variable	category	Mean	t/F stat.	P value	Mean	t/F stat.	P value	Mean	t/F stat.	P value
Gender	Male	90.0 (17.6)	5.064	<0.001	89.2 (17.6)	3.168	0.002	91.4 (17.7)	4.077	<0.001
	Female	83.9 (18.6)			84.1 (18.8)			83.7 (18.5)		
Age	≤ 20	85.6 (18.8)	0.081	0.935	86.6 (18.6)	1.381	0.168	84.4 (19.0)	-1.302	0.193
	>20	85.5 (18.3)			84.4 (18.5)			86.4 (18.1)		
Marital status	Single	85.4 (18.7)	-1.025	0.306	85.8 (18.8)	0.425	0.671	84.9 (18.6)	-1.736	0.083
	Married	87.4 (17.1)			84.6 (14.5)			89.3 (18.6)		
Working Status	Working	90.7 (20.1)	2.787	0.005	86.2 (21.2)	0.169	0.866	94.6 (18.5)	3.711	<0.001
	Not Working	85.1 (18.4)			85.7 (18.3)			84.5 (18.4)		
Smoking	Non-Smoker	85.6 (18.4)	0.683	0.495	85.8 (18.4)	0.332	0.701	85.5 (18.4)	0.565	0.572
	Smoker	83.9 (21.8)			84.4 (22.1)			83.5 (21.9)		
Exercise	No Exercise	82.7 (18.9)	11.104	<0.001 a	84.0 (18.9)	1.640	0.195	81.6 (19.0)	14.286	<0.001 d
	≤ 3 times/week	86.8 (17.3)			87.1 (18.0)			86.4 (16.6)		
	> 3 times/week	89.0 (19.1)			86.0 (19.0)			92.3 (18.7)		
Abused	Yes	78.3 (20.0)	34.160	<0.001 b	78.7 (21.5)	19.146	<0.001 e	78.0 (18.6)	16.158	<0.001 f
	No	88.5 (17.1)			88.8 (16.8)			88.2 (17.5)		
	Not Sure	81.8 (19.3)			79.2 (17.6)			84.4 (20.7)		
Income (SAR)	< 3,000	84.1 (20.8)	7.143	<0.001 c	84.4 (20.8)	7.842	<0.001 g	83.6 (20.9)	1.160	0.314
	3,000-12,000	83.5 (18.7)			82.6 (18.4)			84.5 (19.4)		
	> 12,000	87.8 (18.6)			89.1 (17.2)			86.6 (17.1)		

a LSD No exercise vs ≤3 times/week, and vs >3 times/week, (P<0.001, P<0.001)

b LSD Yes vs No (P<0.001), No vs Not Sure, (P<0.001)

c LSD <3000 SAR vs >12000 SAR (P=0.019), 3000-12000 vs >12000SAR (P<0.001), >12000 vs 3000-12000 (P<0.001)

d LSD No exercise vs ≤3 times/week and vs >3 times/week (P=0.005, P<0.001), and ≤3 times/week vs >3 times/week (P=0.007)

e Yes vs No (P<0.001), and Not Sure vs No (P<0.001)

f Yes vs No (P<0.001), Yes vs Not Sure(P=0.032)

g <3000 vs >12000 (P=0.033), and >12000 vs 3000-12000 (P<0.001)In a multiple regression analysis of 1,148 students, several predictors were found to have a significant effect on flourishing. Well-being emerged as the strongest predictor with a standardized coefficient of 0.50 (p<0.001). External factors as well as disposition and behavior followed with coefficients of 0.20 (p<0.001) and 0.16 (p<0.001), respectively. Gender was positively associated with flourishing, although it had a smaller effect (beta =0.10, p<0.001), whereas religion had a negative influence (Beta=-0.11, p<0.001). Furthermore, employment had a positive effect on flourishing compared to unemployment (beta =0.04, p=0.019), and higher income (>12,000 SAR) slightly increased flourishing (Beta=0.04, t=2.15, p=0.032) (**Table 5**).

**Table 5:** Regression analysis of factors influencing flourishing levels among all students (n=1148).

Variable	Unstandardized coefficient		Standardized coefficient	t-value	Sig.	*R^2^*	*F*	Adj R2
	B	SE	Beta					
Constant	13.99	2.504	-	5.59	< 0.001	0.673	335.58	0.671
Well-being	0.80	0.04	0.50	19.80	< 0.001			
External Factors	0.45	0.05	0.20	8.97	< 0.001			
Disposition and Behaviour	0.75	0.108	0.16	6.97	< 0.001			
Gender (ref: Female)								
Male	3.98	0.717	0.10	5.54	< 0.001			
Religion	-0.68	0.118	-0.11	-5.79	< 0.001			
Working Status (Ref: Not Working)								
Work	2.73	1.156	0.04	2.36	0.019			
Income (Ref: <3000 SAR)								
>12000 SAR	1.36	0.634	0.04	2.15	0.032			

In a multiple regression analysis of 586 college health students, several predictors had a significant effect on flourishing. Well-being was the strongest predictor, with a standardized coefficient of 0.49 (p<0.001), followed by external factors with 0.21 (p<0.001), disposition, and behavior (p < 0.18 (p<0.001). Gender was positively associated with flourishing, although with a smaller effect size (β =0.09, p<0.001), whereas religion had a negative influence (β =-0.08, p=0.002). Interestingly, income between SAR 3,000 and SAR 12,000 was negatively associated with flourishing compared to income below SAR 3,000 (β = -0.06, p = 0.010) (**Table 6**).

**Table 6:** Regression analysis of factors influencing flourishing levels among health college students (n=586) and non-health college students (n=562).

		Unstandardized coefficient		Standardized coefficient	t-value	Sig.	*R^2^*	*F*	Adj R2
College		B	SE	Beta					
**Health**	Constant	14.95	3.57	-	4.191	<0.001	0.672	197.67	0.669
	Well-being	0.77	0.06	0.49	14.101	<0.001			
	External Factors	0.45	0.07	0.21	6.550	<0.001			
	Disposition and Behaviour	0.82	0.16	0.18	5.214	<0.001			
	Gender (ref: Female)								
	Male	3.42	0.96	0.09	3.561	<0.001			
	Religion	-0.52	0.17	-0.08	-3.072	0.002			
	Income (Ref: <3000 SAR)								
	3000-12000 SAR	-2.37	0.91	-0.06	-2.597	0.010			
**Non-health**	Constant	15.72	3.60	–	4.363	<0.001	0.684	149.83	0.680
	Well-being	0.76	0.06	0.47	12.339	<0.001			
	External Factors	0.46	0.07	0.21	6.149	<0.001			
	Disposition and Behaviour	0.79	0.15	0.17	5.287	<0.001			
	Gender (ref: Female)								
	Male	3.91	1.13	0.09	3.461	<0.001			
	Religion	-0.76	0.17	-0.12	-4.540	<0.001			
	Working Status								
	Work	4.52	1.60	0.07	2.825	0.005			
	Abused (Ref: No)								
	Yes	-2.66	1.07	-0.06	-2.486	0.013			
	Exercise	0.43	0.22	0.05	1.979	0.048			

In the multiple regression analysis of 562 students from non-health-related universities, well-being emerged as the most important predictor of wealth, with a standardized coefficient of 0.47 (p<0.001). This was followed by external factors (β =0.20, p<0.001), disposition, and behavior (β =0.17, p<0.001). Gender had a positive, albeit modest, association (β =0.09, p<0.001), whereas religion had a negative effect on flourishing (β =-0.12, p< 0.001). Employment status was positively associated with well-being (β =0.07, p=0.005) and experiencing abuse had a negative impact (β =-0.06, p=0.013). Exercise had a smaller positive effect (β =0.05, p=0.048) (**Table 6**).

## Discussion

In the context of positive psychology, flourishing refers to the overall state of optimal mental health and well-being, characterized by feelings of engagement, meaning, purpose, and fulfillment in life. It goes beyond the absence of mental illness and focuses on thriving and flourishing in various aspects of life. When comparing the level of flourishing among healthy and non-healthy college students, it is important to consider how their academic pursuits, personal experiences, and overall well-being contribute to their sense of flourishing (15). In the current study, college students generally had a high level of flourishing regardless of their field of study. The flourishing score represents an individual’s self-perceived success in important areas, such as relationships, self-esteem, purpose, and optimism. A higher flourishing score indicated a greater sense of well-being and psychological prosperity. This result is consistent with a previous study among 424 undergraduate Filipino students, where flourishing levels were positively linked with students’ academic achievement, positive affect, and life satisfaction (26).

It is important to note that the flourishing scores may vary across studies and populations. For example, a study conducted among Canadian adolescents found that 41% of students reported flourishing scores below the mean, indicating a lower level of well-being in this sample. Furthermore, a scoping review of the measurement of flourishing suggests that collecting information about the methodology, conceptualization, and validation of flourishing scales can help interpret past literature and develop a more cohesive understanding of flourishing in the future (27). Another study conducted among undergraduate nursing students reported flourishing scores from 24.0 to 100.0, with an average of 74.2. Factors such as well-being, disposition, behavior, and external factors are positively correlated with the flourishing index (20).

Moreover, the results illustrated significant differences in flourishing levels based on gender, employment status, exercise, abuse experience, and income. Males showed higher flourishing than females in all groups. This result is inconsistent with that of previous studies (28, 29). For instance, in Spain, female university students reported higher flourishing levels than males (28). The finding of higher flourishing levels among males than females suggest a gender difference in perceptions of well-being and psychological prosperity. It is important to note that this difference may be influenced by various factors including societal norms, cultural expectations, and individual experiences.

Similar studies have explored gender differences in flourishing levels among college students. For example, a study examining the social climate of undergraduate physics courses found that gender differences in course belonging may exist, indicating that females may feel relatively marginalized in the classroom (30). Furthermore, studies have explored gender differences in perceived stress and coping strategies among college students, indicating the need for educational interventions to develop effective coping strategies for both males and females (4). It is important to consider that gender differences in flourishing levels may vary across different populations and contexts. Further research is needed to gain a comprehensive understanding of the factors contributing to these differences.

As per the results of the current study, working participants recorded higher well-being than non-working individuals, with significance in both overall and non-health categories. The finding of higher well-being among working participants suggests a positive association between employment status and well-being among the college students. Employment may contribute to a sense of purpose, financial stability, and social connections, positively impacting overall wellbeing. This finding is supported by other studies, (31, 32), in which a positive association between flourishing and workplace support was confirmed. Similar studies explored the relationship between employment status and well-being among young individuals. For example, a study examining the employment-well-being relationship found that employed young individuals had higher life satisfaction levels than did unemployed individuals. Furthermore, research on the well-being of students in higher education has highlighted the importance of factors that impact student well-being, including employment status (33).

Several significant findings were observed in this study. Participants who exercised more than three times per week demonstrated higher flourishing scores. This suggests that regular exercise may positively affect college students’ academic performance and overall success. Physical exercise enhances individual well-being (34). Additionally, participants who reported abuse had lower flourishing scores. This finding highlights the detrimental effects of abuse experience on college students’ academic success and well-being. This result is in line with a previous study in the United States of 54 universities (35). Furthermore, a higher income was associated with higher flourishing scores, particularly in health colleges. This result is consistent with that of a previous study of 167 participants from the Midwestern U.S. city of Cleveland (36). A possible explanation for our findings is that financial well-being may contribute to students’ academic success, particularly in specific fields of study.

The positive association between exercise frequency and success score suggests that regular physical activity can have beneficial effects on academic performance. Exercise has been linked to improved cognitive function, increased self-control, and reduced psychological distress, which may contribute to better academic outcomes (37). However, the negative impact of experiences of abuse on success scores highlights the need for support and intervention among students who have experienced abuse. Abuse can have various negative consequences including psychological distress, impaired academic functioning, and decreased well-being (38).

The association between higher income and flourishing scores suggests that financial stability and resources positively influence academic performance. Financial well-being can alleviate stressors related to basic needs, provide access to educational resources, and support students’ overall well-being (39).

This study identified several predictors of flourishing. Well-being emerged as the strongest predictor. This finding suggests that individuals with better well-being are more likely to experience flourishing. This result agrees with a study that reported the significant role of social, psychological, and emotional well-being in the flourishing of first-year university students (40). Well-being encompasses various aspects of an individual’s life including relationships, self-esteem, purpose, and optimism. External factors also have a significant impact on flourishing, indicating that external factors such as social support, environmental conditions, and access to resources contribute to an individual’s ability to flourish. Disposition and behavior were also found to be significant predictors of flourishing. This suggests that an individual’s personal traits, attitudes, and behaviors play a role in their ability to experience flourishing. The findings highlight the importance of well-being, external factors, and personal disposition and behavior in predicting flourishing among college students.

Similar studies have explored predictors of flourishing among college students. For example, one study examined the level and prevalence of flourishing among different student subgroups and identified proxy variables for the elements of flourishing as predictors (41). Another study found that academic engagement, positive emotions, and life satisfaction were positively linked to students’ flourishing (26). It is important to consider that predictors of flourishing may vary across studies and populations. Factors such as the cultural context, sample characteristics, and measurement instruments can influence the results.

Although this study reported significant findings, several limitations need to be acknowledged. The cross-sectional design, recruitment of participants from a single institution, and reliance on electronic self-reported questionnaires impact the generalizability of the results, do not establish a cause-and-effect relationship, and may introduce a response bias. Furthermore, the response rate of 51.7% may constrain the representativeness of the sample and contribute to potential selection bias. Future research should aim for higher response rates and include participants from multiple institutions throughout Saudi Arabia to enhance generalizability and mitigate bias. Additionally, while our study identified significant predictors of flourishing, future investigations could benefit from employing advanced analytical techniques, such as Structural Equation Modeling (SEM), to further explore these complex relationships and to provide a more nuanced understanding of how the identified factors interact with one another.

Based on the results of this study, we propose the following implications for enhancing students’ flourishing levels: emphasis should be placed on the early assessment of students’ conditions since they joined the university. University services should focus on providing mental, social, monetary, and living arrangements, specifically for high-risk students. Future research should explore other factors that may be related to student flourishing. Intervention studies should be the next step in planning programs to promote flourishing processes among university students.

## Conclusion

This study aimed to determine the level of flourishing among healthy and non-healthy college students and determine their flourishing predictors. The results show that, although there was no difference in the flourishing level between different colleges, significant differences were found based on gender, employment status, exercise, and income. Several flourishing predictors were identified: well-being, social support, environmental conditions, access to resources, disposition, and behavior. Hence, decision-makers at academic institutions advocate supportive services that focus on enhancing the flourishing of university students.

## Data Availability

The raw data supporting the conclusions of this article will be made available by the authors, without undue reservation.
